# Evaluation of Chemotherapeutic Compounds in the Reticuloses*

**DOI:** 10.1038/bjc.1958.40

**Published:** 1958-09

**Authors:** Edith Paterson


### EVALUATION OF CHEMOTHERAPEUTIC COMPOUNDS IN THE RETICULOSES*

EDITH PATERSON

*From the Christie Hospital, Manchester, England*

Received for publication July 14, 1958

THE proper development of chemotherapy in the management of the malignant reticuloses requires that comparisons are made between various compounds, and with other methods of treatment.

Before a compound reaches the point of clinical trial a great deal of thought and work has been already expended. We are not here concerned with the starting points of those trains of thought that lead to the synthesis of a substance. Synthesis is followed by experimental work on animals and animal tumours and in these tests a compound may show promise. At this point the clinician who is forever seeking compounds of value will willingly but cautiously put it to clinical test. He owes it to his patients to do so ; he also owes it to the experimental worker to give an objective opinion, on the basis of which further work may be either extended or abandoned.

This paper is concerned with this final phase, that of clinical trial and methods of comparison as these have been applied to some common agents. It is intended primarily for the experimental worker to demonstrate not only the limits of our ability to discern the consequences of clinical trial but also to show some of the problems in the clinical approach.

Any assessment of the efficiency of a new chemotherapeutic agent must be based against standard treatments. These should include not only well tested compounds but also irradiation methods where these are applicable.

It is important to emphasise that to-day the clinical aim is still palliation for the vast majority of patients. Cure can only be hoped for in a small number of the strictly localised lymphomas and in these it is a radiotherapy achievement. So with palliation as the immediate objective we are concerned with assessing the length and quality of a remission, the freedom from toxic side effects, and not least the general convenience of the patient.

At the start, therefore, there is the task of defining the term " remission ". It would be academically sound to measure the remission as the number of weeks after treatment until the patient is as a whole no better than he was at the beginning. This is a workable concept in the assessment of remission in solid types of reticuloendothelial disease. It is not a desirable definition for the chronic leukaemias who are usually re-treated before such a state occurs. So for the chronic leukaemias I propose to regard a remission as ending when the clinical evidence shows clearly that the patient is deteriorating. The indices of this include a significant fall in haemoglobin and rise in the primitive cells of the peripheral blood even although neither symptom has deteriorated to pretreatment levels.

* Paper given July 11th, 1958, at 7th International Cancer Congress, Festival Hall, London.

It is possible to state at any stage in the observation of a treated patient that a symptom is improving or is stationary or is progressing, relative to the same symptom before treatment. Such a statement can be given a numerical figure. The average of these figures for all the symptoms constitutes a " clinical index " which sums up the condition of the patient and hence measures the quality of remission. An index which falls to a certain limit can then be used to define the end of a remission.

TABLE I.—*Clinical Indices—Case of Myeloid Leukaemia*

| | 4 months | 12 months |
|---|---|---|
| Nodes: Neck, Axilla, Groin, Other | — | — |
| Spleen | 2 | 2 |
| Liver | — | — |
| Bone marrow | 2 | 1 |
| Haemoglobin | 2 | 2 |
| W.B.C. | 2 | 1 |
| Primitive cells | 2 | 1 |
| Weight | 2 | 0 |
| Well-being | 2 | 1 |
| Ability to work | 2 | 1 |
| | Index $= \frac{16}{8} = 2 \cdot 0$ | Index $= \frac{9}{8} = 1 \cdot 1$ |

Table I shows a number of symptoms so assessed in a case of chronic myeloid leukaemia at 4 months and 12 months after treatment. Opposite each symptom is a number. The code is " 2 " for improvement, " 1 " for an unchanged symptom, and " 0 " for an advancing symptom. A dash means either that a symptom was not present or alternatively that the clinician forgot to annotate it. The average of these figures at 4 months was 2·0. With this mean value the patient was vastly improved. At 12 months, however, the average was 1·1 and the remission was ending.

TABLE II.—*Clinical Indices—Case of Hodgkin's Disease*

| | 2 months | 4 months |
|---|---|---|
| Superficial nodes: Neck —, Axillae 2, Groins 1 | 1·5 | 1·0 |
| Mediastinum | — | 0 |
| Spleen | 1 | 1 |
| Liver | — | — |
| Effusions | — | — |
| Haemoglobin | 2·0 | 2·0 |
| Fever | 2·0 | 0 |
| Well-being | 2·0 | 1·0 |
| Weight | 2·0 | 1·0 |
| Ability to work | 2·0 | 1·0 |
| | Index $= \frac{12 \cdot 5}{7} = 1 \cdot 8$ | Index $= \frac{7}{8} = 0 \cdot 88$ |

Table II shows a similar study of a case of Hodgkin's disease. The mean index at 2 months was 1·8 ; the patient was much improved. At 4 months, however, the figure was below 1·0 and the remission was over.

The criteria on which the figures are chosen are important but they are too detailed to describe here. Other criteria might well be used. The only measure of validity is that the index arrived at should agree with a clinical impression of the patient as a whole.

The method is not intended to indicate when treatment should be re-instituted —this depends on many other factors. It is only a simple way of expressing the total clinical impression for assessment purposes, in a way which will sort out a welter of symptoms and so facilitate a tabulation of comparative figures on an objective basis. It will not only give us a figure for the length of a remission, it will also estimate the amount of benefit received, and, on a closer analysis, it can be used to look at individual symptoms which might be specifically benefited by a particular agent.

The use of the method is best illustrated by examples in which comparisons have been made between different treatments. The first concerns the approach to measuring response in chronic myeloid leukaemia.

Fig. 1.

*Chronic Myeloid Leukaemia*

Fig. 1–5 each summarise the career of a case of chronic myeloid leukaemia treated by various agents. The sequence of events will be familiar to all clinicians but perhaps less so to those working in the experimental field. These histories are being used to illustrate what I think to be a general truth, that only the first treatment of a leukaemic patient should be used in the comparative assessment of compounds.

The height of the column denotes the period during which the patient was in remission. If no remission occurred during a treatment, that is to say, if the index was 1·0 or less, the column has been barred diagonally.

The patient illustrated in Fig. 1 was given 32P as the first treatment with in consequence a remission of over 2 years. At the end of this remission he was given the chemical compound B.E.P. (1 : 3-bis (ethyleneiminosulphonylpropane)), and a further good remission of 11 months followed. This ended when the patient stopped taking B.E.P. on account of nausea. Myleran was, therefore, substituted. The response was slower and the remission was shorter. The last three courses with mercaptopurine, myleran and a last treatment with 32P were all ineffective. This case illustrates that the duration of remissions steadily diminished and that the same 32P which apparently was most suitable at an early stage, was entirely unhelpful terminally.

This next case (Fig. 2), was given splenic irradiation as the first method of treatment. This patient had a prolonged remission for over three and a half years.

FIG. 2.

FIG. 3.

The treatment was changed to myleran because the spleen seemed to be becoming resistant. This remission lasted for about one year. The patient then deteriorated rapidly.

On the basis of these cases it might seem that myleran was rather ineffectual compared with irradiation methods. Fig. 3 however shows myleran in a very different light. This patient was treated by myleran as the first and only treatment. She was kept in good health for three and a half years and died a few months after the remission ended. It is interesting to note here that at the beginning the amount of myleran required to give freedom from symptoms was small ; later, sustained maintenance dosage was required.

Fig. 4 illustrates the use of urethane in inducing a remission which lasted for over three years. Splenic irradiation resulted in a much shorter remission. Finally mercaptopurine yielded only a transient response.

The last patient, (Fig. 5), was treated by monthly doses of triethylene melamine (T.E.M.) and did well for 16 months. At the end of the remission B.E.P. was given unsuccessfully. X-ray therapy to the spleen was effective for a very short time ; no benefit was obtained with myleran.

These are examples ; similar pictures could be shown of other chemotherapeutic agents. These diagrams all illustrate the principle of diminishing returns. They serve to show how important it is to test compounds in patients at a stage

Fig. 4.

when they are capable of responding and it would seem important to evaluate a new compound on its use as the first agent in treatment.

If this is accepted it follows that very few centres to-day can get an answer about a new treatment quickly, based on the first treatment of their own clinical material. Of recent years the difficulty has further increased because many patients have already been treated by individual physicians before reaching a centre engaged in clinical investigation.

The conclusion is obvious that important comparisons should be done on a national or international basis. This would imply rules for random selection of patients and carefully thought out standards of measurement. For future assessments of chemotherapeutic compounds in chronic myeloid leukaemia it is suggested that splenic irradiation might well be the best control treatment ; this does not mean resurrecting the dusty histories of 20 years ago to compare with a new compound, but rather the selection of contemporary cases for treatment, on a random basis.

Another point is illustrated by these five patients. All had clinical indices of the order of 2 during their first remission ; and in all of them the first treatment yielded a fairly long remission. Yet this rather uniform result was obtained with very diverse agents. One wonders whether all these agents are " specific " or that none is.

*Generalised Hodgkin's Disease*

In the solid reticuloses the use of a compound as the first treatment may be rather less decisive in its effects, than in the leukaemias. Generalised Hodgkin's disease has been used to exemplify the comparison of 2 compounds on the basis of clinical indices. The clinical material comprises 80 section-proved cases, chosen on the sole ground of having each been treated by at least 2 different chemotherapeutic agents, including nitrogen mustard and triethylene melamine which will be

FIG. 5.

compared. Individual cases in this group were also treated with other chemical compounds or irradiation. Such other treatments have been exluded from the present analysis.

These generalised cases have been grouped, depending on the order in which the compounds were given. I am comparing, therefore, nitrogen mustard and T.E.M. where these agents were the first treatments, and the same compounds when these were given as later treatments.

The comparisons are shown in Tables III to VII ; these summarise the relative effects of nitrogen mustard and T.E.M. in terms of :

(*a*) The fraction of responding patients.
(*b*) The length of the response.
(*c*) The quality of the response.
(*d*) Undesirable side effects.

(a) *The fraction of cases responding*

This has been taken as the number of patients whose index was over 1·0 eight weeks or more after treatment. It excludes transitory betterment of shorter duration.

It will be observed that either agent given as a first treatment induced a higher remission rate than if given subsequently. The difference between the 1st and later

TABLE III.—*Comparison between $HN_2$ and T.E.M. in Hodgkin's Disease*

Fraction of patients benefited 8 weeks after treatment

| | Benefited (%) |
|---|---|
| $HN_2$—1st treatment . . . | 80 |
| T.E.M.—1st treatment . . . | 70 |
| | |
| $HN_2$—later treatment . . . | 48 |
| T.E.M.—later treatment . . . | 57 |

treatment with $HN_2$, i.e., between 80 and 48 per cent is significant at the 5 per cent level, and is in line with the leukaemia findings. Nitrogen mustard appears somewhat more successful than T.E.M. as a first treatment, and T.E.M. seems more impressive as a later treatment.

(b) *The length of the response*

This compares the length of remission of the same group of patients who showed betterment at 8 weeks.

TABLE IV.—*Comparison Between $HN_2$ and T.E.M. in Hodgkin's Disease*

Length of remission in weeks

| | Median (weeks) |
|---|---|
| $HN_2$—1st treatment . . . | 22 |
| T.E.M.—1st treatment . . . | 12 |
| | |
| $HN_2$—later treatment . . . | 8 |
| T.E.M.—later treatment . . . | 12 |

The length of remission from $HN_2$ as a first treatment was longer than when T.E.M. was employed. However, for subsequent treatments T.E.M. again seemed to be preferable to $HN_2$.

While both tables appear to favour $HN_2$ as a first treatment, and T.E.M. as a later treatment, the differences are not significant with the numbers available. It is therefore unnecessary to speculate on the interpretation.

(c) *The quality of the response*

This is a comparison of the clinical index of those responding cases, 8 weeks after treatment :

TABLE V.—*Comparison between $HN_2$ and T.E.M. in Hodgkin's Disease*

Clinical index at 8 weeks

| | Mean index—Responding patients |
|---|---|
| $HN_2$—1st treatment . . . . | 1·6 |
| T.E.M.—1st treatment . . . | 1·5 |
| | |
| $HN_2$—later treatment . . . | 1·6 |
| T.E.M.—later treatment . . . | 1·5 |

It would seem that for both first and later treatments nitrogen mustard appeared to be a fraction superior to T.E.M. but the difference if present was very small. The figures also suggest that the quality of a remission was about the same for first and for later treatments with either compound.

(d) *Side effects*

An important consideration in the comparison of two compounds relates to the undesirable side effects. Nitrogen mustard has the well known disadvantage of inducing transient gastrointestinal symptoms and sometimes venous thrombosis ; T.E.M. is given orally in enteric coated tablets and if given in instalments is free of these side effects. This point is conceded and needs no elaboration.

The depression of the haemopoietic system can perhaps be briefly contrasted by examining the effects on two elements of the circulating blood—the haemoglobin and the platelets. The comparative effects of the two compounds on haemoglobin is shown in Table VI which is based on all patients whether they benefited or not provided that the data on their blood counts was available, and irrespective of the order in which compounds were given. Depression of haemoglobin has been taken as a fall of 10 per cent or more following treatment.

TABLE VI.—*Comparison Between $HN_2$ and T.E.M. in Hodgkin's Disease*

Reduction in haemoglobulin—all cases

| | Cases (%) |
|---|---|
| $HN_2$ . . . . | 18 |
| T.E.M. . . . | 23 |

Eighteen per cent of the $HN_2$ patients showed this against 23 per cent of the T.E.M. patients. If this difference were significant it would be important to the clinician. It is, however, not significant, and for both compounds the recovery time of the haemoglobin was the same. There was no difference in the haemoglobin depression between first and later treatments with either compound.

TABLE VII.—*Comparison Between $HN_2$ and T.E.M. in Hodgkin's Disease*

Reduction in platelets

| | Cases (%) |
|---|---|
| $HN_2$ . . . . | 33 |
| T.E.M. . . . | 35 |

The standard measurement of platelet depression was a reduction of 25 per cent or more in the platelet count. The figures of 33 per cent for $HN_2$ and 35 per cent for T.E.M. are very close, and the recovery times were similar for the two compounds. There was no difference between first and later treatments with either compound.

Studies of the other cells of the circulating blood have not shown differences between $HN_2$ and T.E.M.

For the future evaluation of new compounds for generalised Hodgkin's disease a control method of treatment is required. Nitrogen mustard itself would be suitable, on the grounds of the vast clinical experience which has been accumulated.

These clinical comparisons do not have the elegance of laboratory experiment. For example nitrogen mustard and T.E.M. have been compared on animal tumours, notably by Druckrey *et al.* (1956) and by Oettel and Wilhelm (1957). Nevertheless it is of interest that for the Yoshida ascites tumour in rats, these authors have shown comparisons which resemble the clinical findings in Hodgkin's disease.

The differences between the two compounds for other animal tumours should stimulate interest in seeking differences in effect which may exist for other human tumours.

### *Localised Hodgkin's Disease*

Finally here is a further brief illustration of measurement. There is a justifiable hypothesis that two agents in combination might be more effective. This may be based on the idea of synergism as exemplified by the combination of mercaptopurine with azaserine in acute leukaemia. Or it may be based on the idea that a systemic agent might control small undetected foci of disease while a local agent such as X-rays eradicated a mass.

Some time ago we tested this second idea in *early* cases of Hodgkin's disease still apparently localised to one region—neck or axilla. We compared cases treated with regional x-irradiation alone with cases treated by regional x-irradiation plus a full dose of nitrogen mustard given as part of the first planned treatment. If the hypothesis was sound this would be shown as a lengthening of the survival time of the patient or at least as a lengthening of the first remission. The results are shown in Tables VIII and IX.

TABLE VIII.—*Comparison Between $HN_2$ + X-rays with X-rays Given Alone*

Localised Hodgkin's disease
The 5-year survival

| | Number treated | Crude % survival |
|---|---|---|
| Treated by $HN_2$ + X-ray | 35 | 54 |
| Treated by X-ray alone | 32 | 59 |

TABLE IX.—*Comparison Between $HN_2$ + X-rays with X-rays Given Alone*

Localised Hodgkin's disease
Length of 1st remission (assessed at 5th year)

| | Median (months) |
|---|---|
| $HN_2$ +X-ray | 46 |
| X-ray alone | 50 |

The differences shown in Tables VIII and IX are not significant and they do not support the hypothesis that the combined treatment carried any advantage.

A similar examination based on other types of malignant disease or with other compounds might not lead to the same conclusion. This study applies only to Hodgkin's disease and it is included to show that the method of measurement must vary with the information one is looking for.

### SUMMARY

(1) The present-day needs for the advancement of chemotherapy include critical comparisons using defined clinical indices.

(2) The most valuable comparisons are between the effects of different agents as the first treatment and using the average length of remission as the standard.

This requires collaborative work between centres in order to get information that is speedy and significant.

(3) Other effects such as the remission rate or the quality of a remission can equally be assessed by the use of a clinical index, and for certain purposes the survival time may be useful.

(4) For each disease studied a well tested method of treatment is required as a control for a new compound. This implies that treatments should be carried out on a random basis.

### REFERENCES

DRUCKREY, H., SCHMAHL, D., DANNEBERG, T., KAISER, K., NIEPER, H. A., LO, H. W. AND MECKE, R.—(1956) *Arzneimittelforschung*, **6**, 539.

OETTEL, H. AND WILHELM, G.—(1957) *Arch. exp. Path. Pharmak.*, **230**, 559.


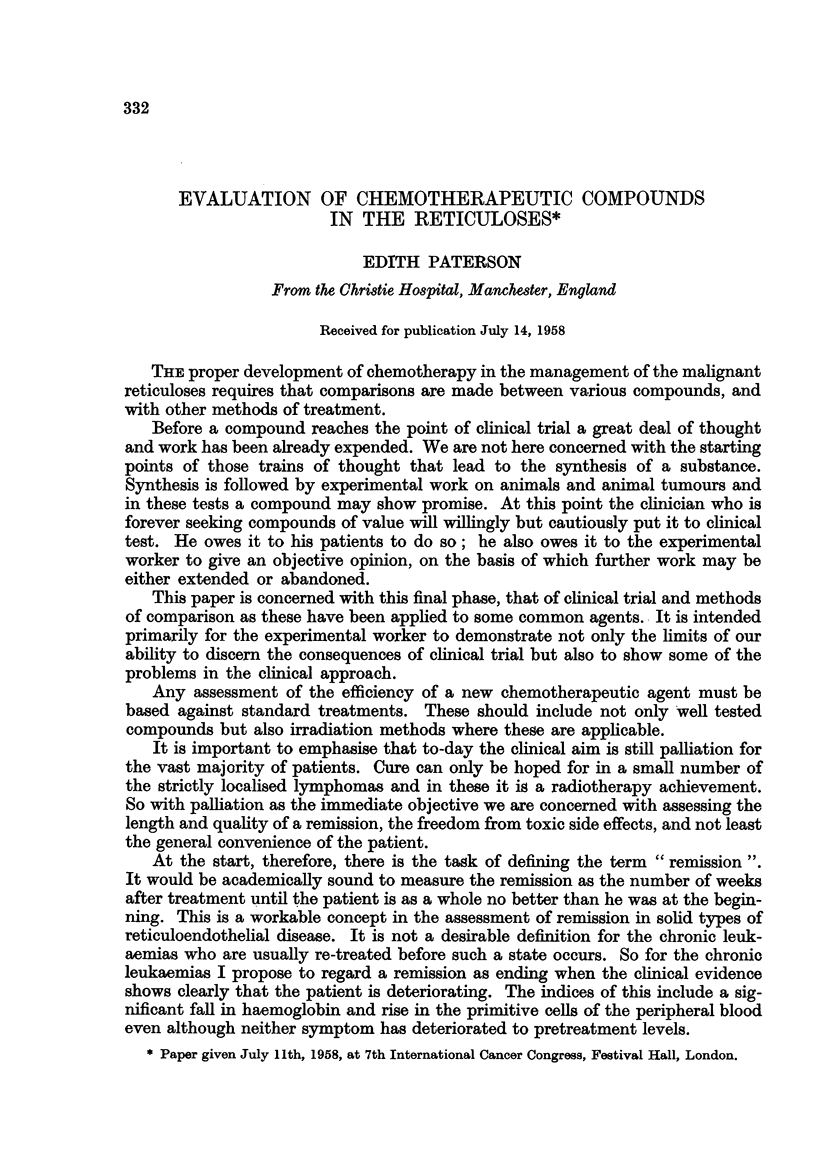

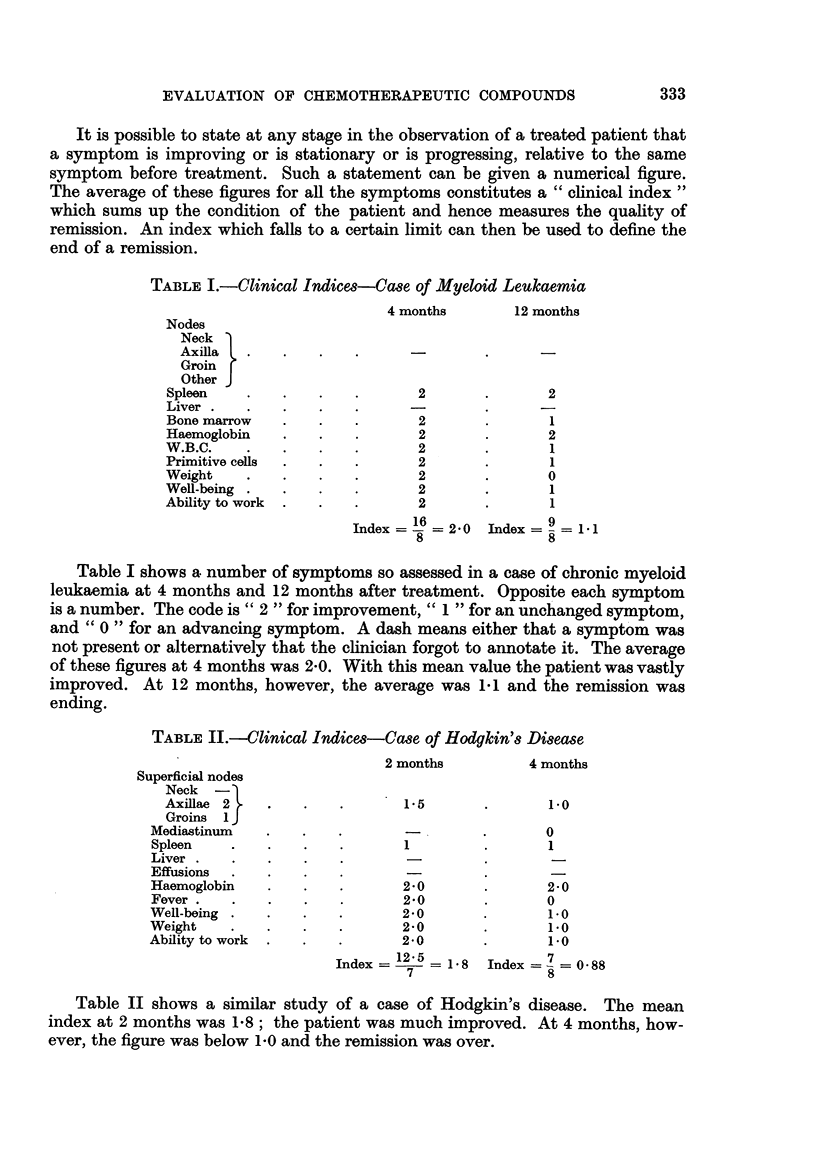

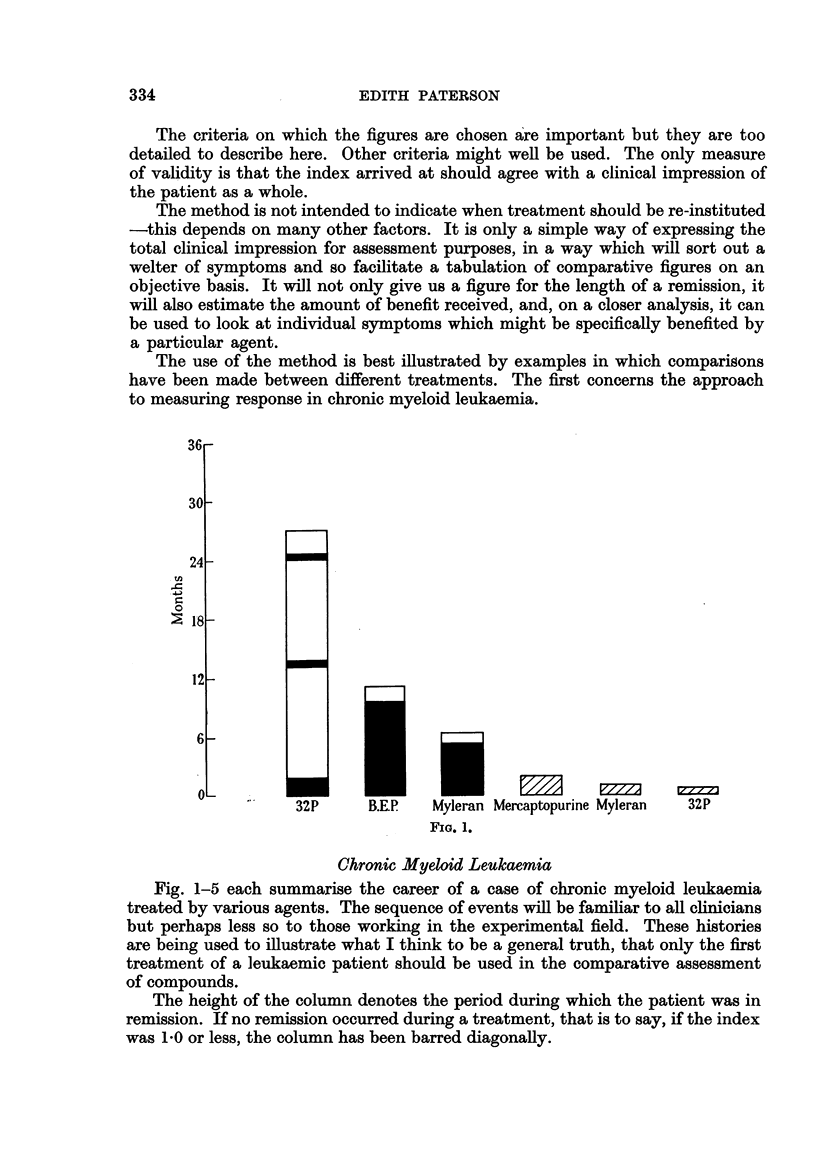

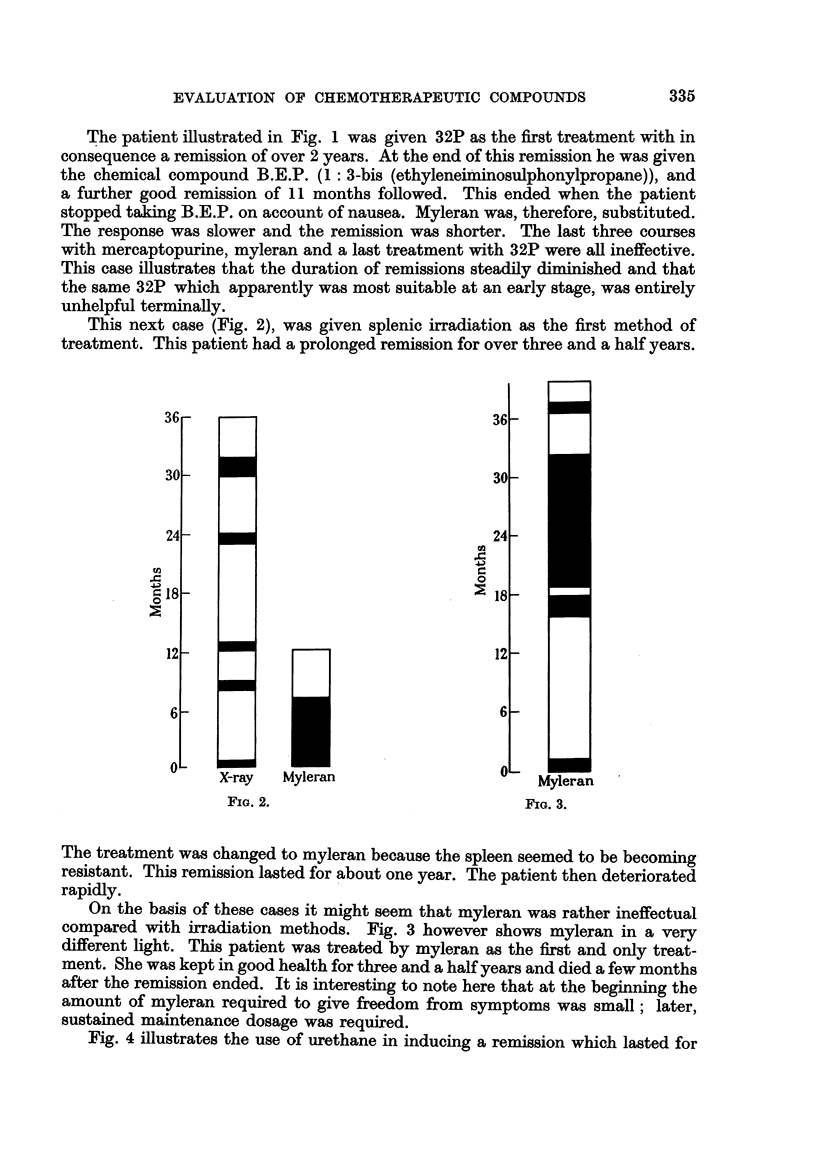

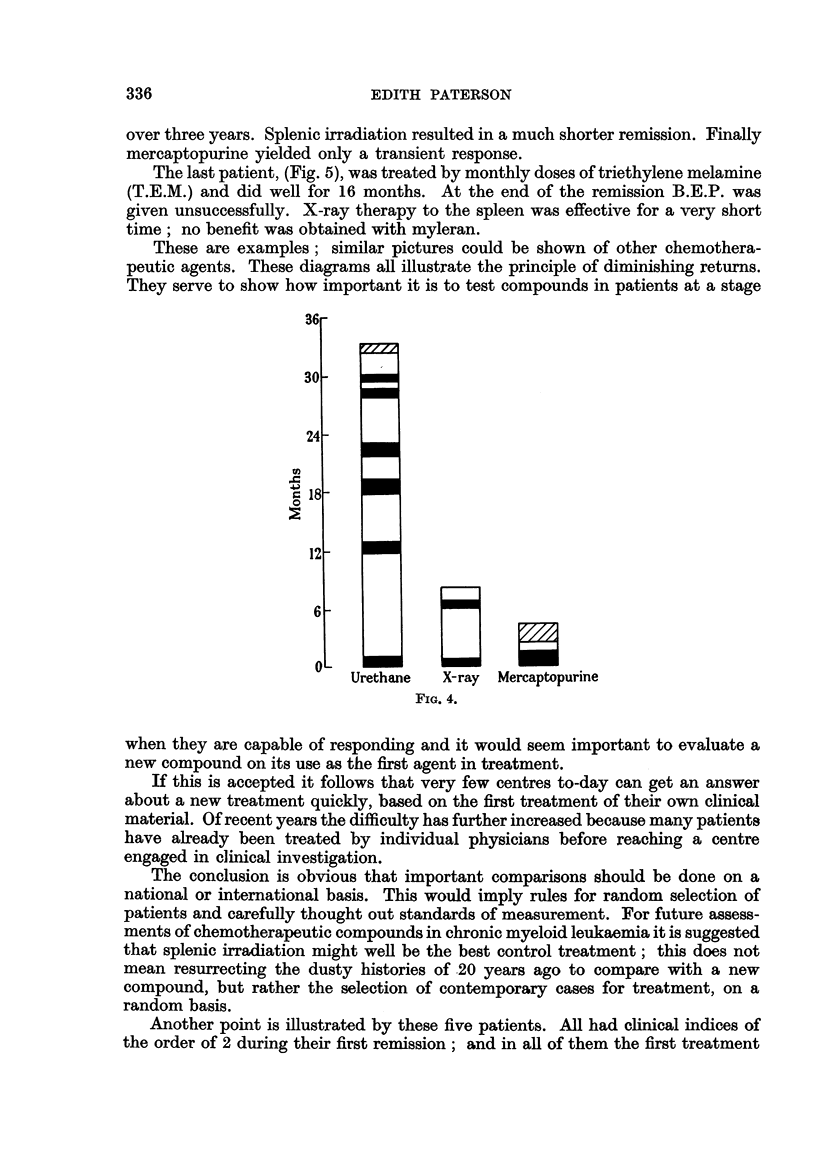

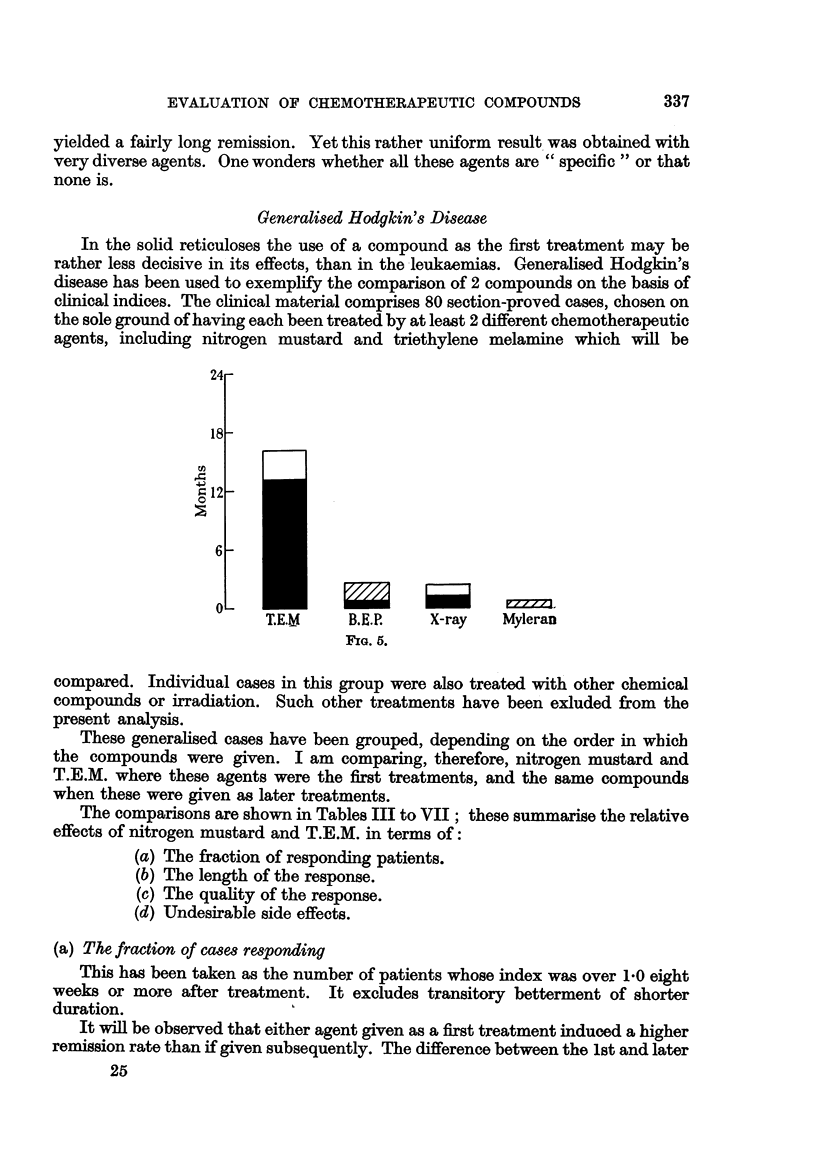

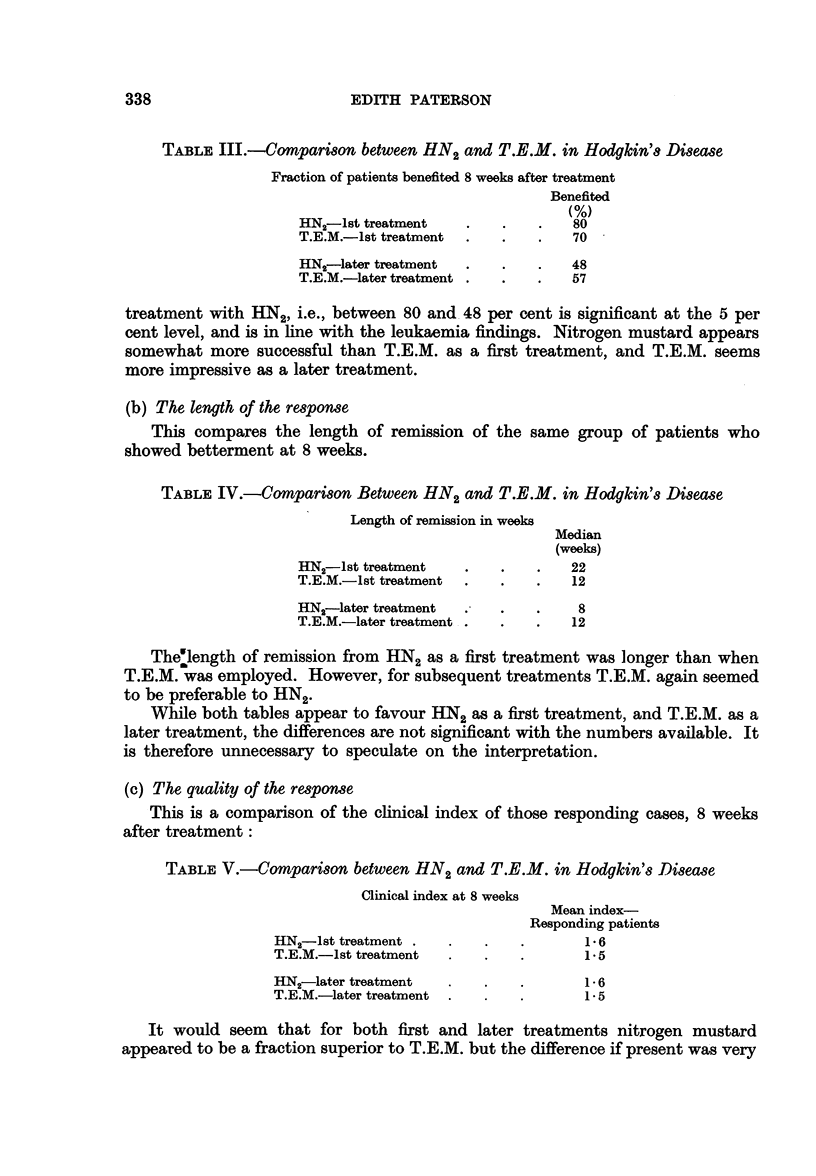

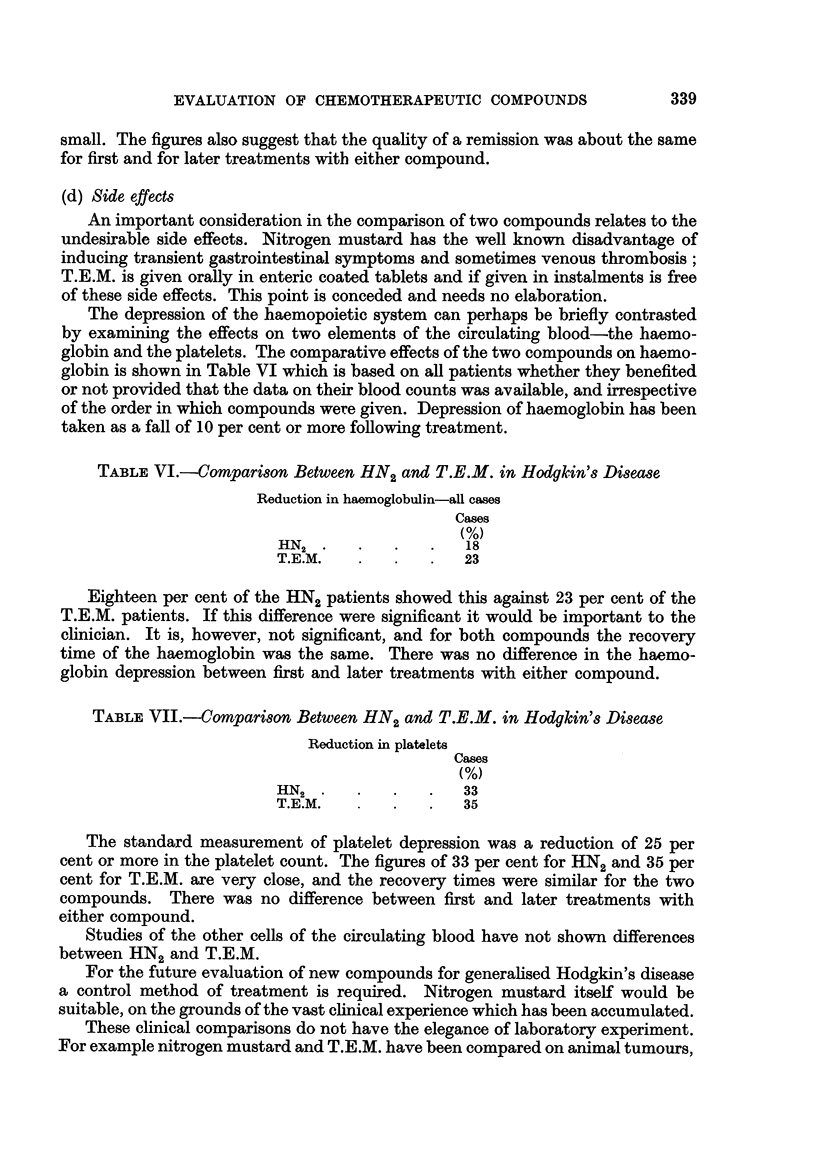

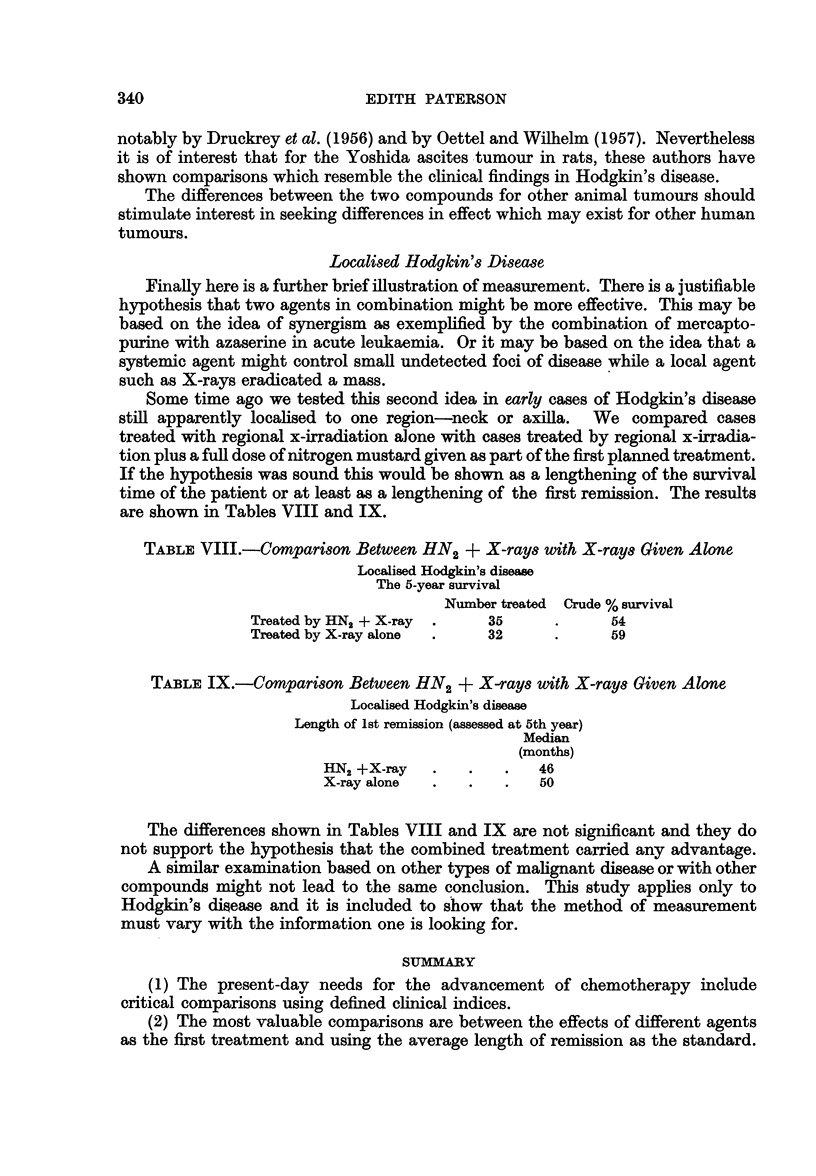

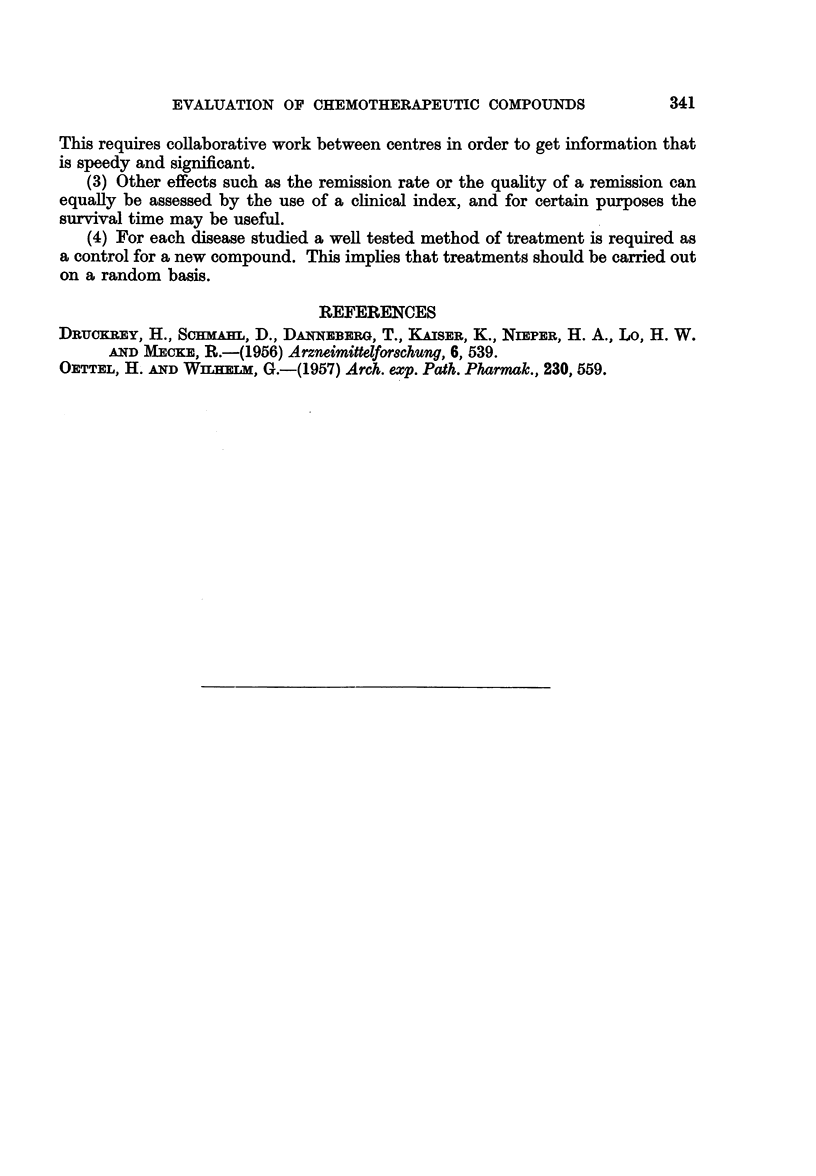


## References

[OCR_00621] DANNEBERG P., DRUCKREY H., KAISER K., LO H. W., MECKE R., NIEPER H. A., SCHMAHL D. (1956). Vergleichende Prüfung der chemotherapeutischen Wirkung von N-oxyd-Lost und anderen alkylierenden Substanzen auf Tumoren von Ratten.. Arzneimittelforschung.

[OCR_00625] OETTEL H., WILHELM G. (1957). Vergleichende Prüfung von 14 cytostatisch wirksamen Produkten an 7 Tiertumoren.. Naunyn Schmiedebergs Arch Exp Pathol Pharmakol.

